# Favorable outcomes with reduced steroid use in juvenile dermatomyositis

**DOI:** 10.1186/s12969-021-00615-0

**Published:** 2021-08-17

**Authors:** Amir B. Orandi, Lampros Fotis, Jamie Lai, Hallie Morris, Andrew J. White, Anthony R. French, Kevin W. Baszis

**Affiliations:** 1 Division of Pediatric Rheumatology/Immunology, Washington University of School of Medicine, St Louis, MO USA; 2 Present Address: Department of Pediatric and Adolescent Medicine, Division of Pediatric Rheumatology, Mayo Clinic, Rochester, MN USA; 3 Present Address: Department of Pediatrics, Division of Pediatric Rheumatology, National and Kapodistrian University of Athens, Athens, Greece; 4 Department of Pediatrics, Washington University School of Medicine, St. Louis, MO USA; 5 Present Address: Department of Pediatrics, Division of Pediatric Rheumatology, Baylor College of Medicine, Houston, TX USA; 6 Present Address: Division of Neonatology, George Washington University School of Medicine and Health Science, Washington, D.C, USA

**Keywords:** Juvenile dermatomyositis, Calcinosis, Biologic therapy, Glucocorticoids, Pediatric rheumatology

## Abstract

**Background:**

High-intensity glucocorticoid regimens are commonly used to induce and maintain remission in Juvenile Dermatomyositis but are associated with several adverse side-effects. Evidence-based treatment guidelines from North American and European pediatric rheumatology research societies both advocate induction with intravenous pulse steroids followed by high dose oral steroids (2 mg/kg/day), which are then tapered. This study reports the time to disease control with reduced glucocorticoid dosing.

**Methods:**

We retrospectively reviewed the records at a single tertiary-care children’s hospital of patients diagnosed with Juvenile Dermatomyositis between 2000 and 2014 who had a minimum of 2 years of follow-up. The primary outcome measure was time to control of muscle and skin disease. Additional outcome measures included glucocorticoid dosing, effect of treatment on height, frequency of calcinosis, and complications from treatment.

**Results:**

Of the 69 patients followed during the study period, 31 fulfilled inclusion criteria. Median length of follow-up was 4.58 years, (IQR 3–7.5). Myositis control was achieved in a median of 7.1 months (IQR 0.9–63.4). Cutaneous disease control was achieved in a median of 16.7 months (IQR 4.3–89.5). The median starting dose of glucocorticoids was 0.85 mg/kg/day, (IQR 0.5–1.74). The median duration of steroid treatment was 9.1 months, (IQR 4.7–17.4), while the median duration of any pharmacotherapy was 29.2 months (IQR 10.4 to 121.3). Sustained disease control off medications was achieved in 21/31 (68%) patients by the end of review. Persistent calcinosis was identified in only one patient (3%).

**Conclusion:**

Current accepted treatment paradigms for Juvenile Dermatomyositis include oral glucocorticoids beginning at 2 mg/kg/day and reduced over a prolonged time period. However, our results suggest that treatment using reduced doses and duration with early use of steroid-sparing agents is comparably effective in achieving favorable outcomes in Juvenile Dermatomyositis.

## Background

Juvenile Dermatomyositis (JDM) is a rare inflammatory myopathy in children, comprising 85% of all idiopathic inflammatory myopathies of childhood [1]. It is a chronic immune-mediated vasculopathy associated with proximal muscle weakness, characteristic skin involvement, and impairment in physical function [2]. Diagnosis has long been based on the clinical and laboratory criteria of Bohan and Peter [3, 4]⁠, but new classification criteria have been recently developed incorporating weighted scores for clinical features of characteristic cutaneous changes, symmetric proximal muscle weakness, elevated serum muscle enzymes, myopathic changes on electromyogram, and characteristic muscle biopsy abnormalities, combined with absence of histopathologic signs of other myopathies [5]. The outcome of patients with JDM prior to the 1960s was poor, as more than one-third of patients died from their illness and one-third developed permanent limitations [6, 7]. Following the introduction of glucocorticoids, which became a mainstay of the treatment in JDM, mortality rates declined. After increased use of other immunomodulatory agents such as methotrexate and azathioprine [8, 9], the mortality rate further declined to estimates of less than 2–3% [10, 11].

During this era of gradual improvement in treatment outcomes, there were no randomized controlled trials or published corticosteroid treatment regimens to guide therapeutic decisions. In fact, only recently has a randomized controlled trial studied superiority of two different immunomodulatory agent in combination with prednisone to prednisone alone; however, this manuscript was published in 2016 many years after practice trends had developed [12]. Generally, different doses, routes, and duration of glucocorticoids were first-line therapy for mild, moderate, and severe JDM in combination with other immunomodulatory agents. However, a number of published reports advocated that initial high-dose, intravenous and/or pulsed intervals of glucocorticoids were needed to aggressively suppress disease activity, sustain remission, and prevent calcinosis, a highly morbid JDM complication [13–17]. Despite this trend, there remained tremendous variety in practice, with most authors advocating an initial oral prednisone dose of 2 mg/kg/day (up to a maximum of 60–80 mg/day), with pulses of high-dose (up to 30 mg/kg/day, maximum 1 g daily) intravenous methylprednisolone (IVMP) for moderate to severe cases as induction therapy, followed by a slow taper of oral steroids over 1 year [18]. However, lower to medium doses of prednisone (1 to 1.5 mg/kg/day) were also reported effective, particularly after taking into account the disability associated with higher glucocorticoid dosing [19]. Another report showed comparable outcomes using combinations of other immunosuppressive agents with *no* use of systemic glucocorticoids, albeit in an admittedly mild disease phenotype where the practice was not universal amongst treated patients [20]. Not only have disease outcomes improved with addition of steroid-sparing agents, but it is recognized that aggressively utilizing these agents can reduce the burden of adverse effects of glucocorticoids without sacrificing outcome [21]. Regardless, the most current recommendations continue to endorse high-dose glucocorticoids: Consensus-based treatment plans published by the Childhood Arthritis and Rheumatology Research Alliance (CARRA) for the initial treatment of moderate to severe JDM recommended 2 mg/kg/day oral prednisone in combination with methotrexate in their most conservative protocol, with the two more aggressive protocols utilizing intravenous methylprednisolone (30 mg/kg/day, maximum 1 g) for 3 days and continuing with weekly or monthly intravenous methylprednisolone pulses [22, 23]⁠. Similarly, European recommendations from the Single Hub and Access point for Pediatric Rheumatology in Europe (SHARE) recommended high-dose intravenous dosing of methylprednisolone (15–30 mg/kg/day, maximum 1 g/day) for all patients at diagnosis or with a disease flare, followed by an oral prednisone taper beginning at 1–2 mg/kg/day [24]. To our knowledge, there have been no reports describing outcomes with lower doses of glucocorticoids used consistently in early combination with other immunosuppressive agents as a universal approach in a single referral center.

Given that high-dose and/or prolonged use of glucocorticoids have many potential adverse events (including growth retardation, hypertension, impaired glucose tolerance, immunosuppression, osteopenia, recurrent infections, vertebral fractures, and avascular necrosis) [25], the approach at our center has been to use the minimum dose and duration of glucocorticoids necessary, in order to induce remission and control disease activity, in combination with early and consistent use of steroid-sparing agents. Here, we report the outcomes of JDM patients treated in this manner at as single tertiary-care children’s hospital during a 14-year period, that coincides with the timeframe of the above referenced publications.

## Methods

### Study population

We retrospectively reviewed the charts of patients with JDM diagnosed and treated at St. Louis Children’s Hospital between January 2000 and December 2014. We included patients with probable or definite JDM, according to the Bohan and Peter criteria [3, 4], with disease onset prior to 18 years of age, and with at least 2 years of subsequent follow up. Patients with mixed connective tissue disease, overlap syndrome, or amyopathic JDM were excluded. Patients were also excluded if they transferred care to our institution after being diagnosed and/or treated at other institutions. This study was approved by the Human Research Protection Office of Washington University School of Medicine in St Louis #201107133.

### Definitions

Symptom onset was defined as the time when patient, parent, guardian, or pediatrician first observed JDM symptoms (e.g., weakness or rash). Myositis control was defined as the time when normal muscle strength (5/5 in all major muscle groups) and normal muscle enzyme levels (creatine kinase, aldolase, lactate dehydrogenase, aspartate aminotransferase, and alanine aminotransferase) were documented. Cutaneous disease control was defined as the time when no active JDM rash was documented on physical exam. Complete disease control was, therefore, defined as achievement of both myositis and cutaneous disease control. Medications were gradually withdrawn after complete disease control was maintained for at least 6 months, but most often 12 months. Once all medications were withdrawn, and muscle and skin disease remained quiescent, the patient was considered to have sustained disease control.

Disease course was defined as 1) monocyclic when the patient had no clinical or laboratory markers consistent with active disease and was off all medications within 24 months of diagnosis, 2) chronic continuous when there was persistent disease or continued treatment with medications for more than 24 months after diagnosis, and 3) polycyclic when there was recurrence of disease after at least 6 months of no clinical or laboratory disease activity. These definitions are similar to those used in prior studies [15]. Initial disease severity was classified as mild, moderate, or severe based on presenting disease features, including degree of muscle weakness, presence of dysphagia or dysphonia, organ involvement, ulcerations, and degree of skin involvement.

### Treatment protocol

Our treatment protocol consisted of a stepwise approach depending on disease severity at presentation and evolution of symptoms following treatment initiation. Upon diagnosis, patients with mild disease were started on oral prednisone and methotrexate. Patients with moderate to severe disease were treated with IVMP, followed by an oral steroid taper, and methotrexate. IVMP was *only* used either as an initial therapy within the first month of diagnosis and/or in cases of severe relapse. Varying schedules and dosages were used for patients receiving IVMP, ranging from 10 to 30 mg/kg/day for up to 3 days, with maximum 1 g/day. The oral steroid taper uniformly began at a dose less than 2 mg/kg/day, with the taper duration dependent on the clinical and laboratory response of each patient, but without specific objective criteria for tapering. Methotrexate was initiated at or within 1 month of diagnosis at a dose of 12–15 mg/m^2^ weekly (primarily subcutaneously). Patients in whom complete disease control was not achieved within 3–6 months, including those who had worsening of disease with steroid weans, were started on monthly intravenous immune globulin (IVIG) at 2 g/kg/dose. Patients with severe disease received IVIG sooner after diagnosis. Hydroxychloroquine was added for those patients with persistent skin disease that did not respond to the above treatments. Alternative agents less commonly used included rituximab, leflunomide, cyclosporine, cyclophosphamide, and mycophenolate mofetil. Treatment was weaned in a stepwise fashion after the patient had remained in complete disease control for 6–12 months.

### Adverse effects of glucocorticoids

Growth parameters were obtained at diagnosis and at subsequent follow up visits, and the z-scores for height and BMI were calculated based on the CDC growth charts for the United States published in 2000 [26]. Other major adverse effects of glucocorticoids, including immunosuppression, recurrent infections, vertebral fractures, and avascular necrosis, were abstracted.

### Statistical analysis

Analysis was performed using SAS Version 9.3 (SAS Institute) and SPSS Version 22.0 (IBM Analytics). Categorical variables were reported as percentages, and continuous variables as medians and interquartile ranges. Time-to-event analyses were conducted with Kaplan-Meier analyses. The starting time was the date of diagnosis, and data from participants not experiencing the outcome were censored at the time of last follow-up. The effect of steroid medication on participant height was modeled with a random-effects mixed-model analysis. Participants were considered a random effect, and years from diagnosis and its square were considered fixed effects. Mann-Whitney test was used for the comparison between the disease groups.

## Results

### Demographic characteristics (Table 1)

Of 69 JDM patients diagnosed and/or treated between 2000 and 2014, 19 patients were excluded due to transfer of care from outside institutions or missing initial diagnostic and management data. Eleven patients had inadequate follow-up for inclusion. Eight patients were excluded due to diagnosis of amyopathic JDM. Therefore, 31 patients (12 males, 19 females) were included in the study. Median duration of follow up of this group was 4.6 years (IQR 3–7.5). The median age of the patients at the time of diagnosis was 8.1 years (IQR 5.6–10.5). The median duration of untreated disease (time from symptom onset to diagnosis and treatment) was 2.9 months (IQR 1.5–8.6).

**Table 1:** Characteristics of patients at the time of diagnosis

**Sex**	12 male (39%)	
	19 female (61%)	
**Age [median, (IQR)]**	8.1 years (5.6–10.5)	
**Weight, kg [median (IQR)]**	25.1 (19.2–36.5)	
**Symptom duration prior to treatment [median, (IQR)]**	2.9 months (1.5–8.6)	
**Muscle enzymes**	**Median (IQR) in U/L**	**Normal range in U/L**
AST (*n* = 26)	57 (47–177)	8–60
ALT (*n* = 26)	48 (27–120)	7–55
CK (*n* = 28)	328.5 (144–1957)	29–308
Aldolase (*n* = 28)	12.3 (8.7–24.2)	< 14.5
LDH (*n* = 16)	773 (510–953)	145–293
**MRI**	Diagnostic in 6 out of 7 patients	
**EMG**	1	
**Muscle Biopsy**	7	
**Myositis antibodies**	12 out of 19 positive (63%)	
Anti P155/140	4 (33%)	
Anti Mi-2	3 (25%)	
Unidentified S35 band	5 (41%)	

Myositis antibody panels were routinely obtained in our center only after 2009. A myositis antibody panel was obtained in 61% of patients (19/31) patients and was positive in 63% (12/19), identifying anti-p140/155 antibody in four patients (33%), anti-MI-2 in three patients (25%), and the presence of an unidentified antibody by S-35 immunoprecipitation in five patients (42%). These latter five cases were obtained prior to 2009.

### Treatment

All but one patient received glucocorticoids (prednisone or prednisolone) at diagnosis, at a median dose of 0.85 mg/kg/day (IQR 0.5–1.74). The maximum daily oral dose was 60 mg, which only five patients (16%) received. All patients were also treated with methotrexate, at a median dose of 13.3 mg/m^2^ (IQR 12–15). Twelve patients (38%) received IVMP within 1 month of diagnosis. Fifteen (48%) were treated with monthly IVIG, 2 g/kg. Five patients (16%) were treated with rituximab, four (13%) with leflunomide, 13 (42%) with hydroxychloroquine, two (6%) with mycophenolate mofetil, four (13%) with cyclophosphamide, three (10%) with anti-tumor necrosis factor (TNF)-alpha agents, and one with cyclosporine. No patients underwent placement of central lines for intravenous medications.

### Outcomes

**Fig. 1:**
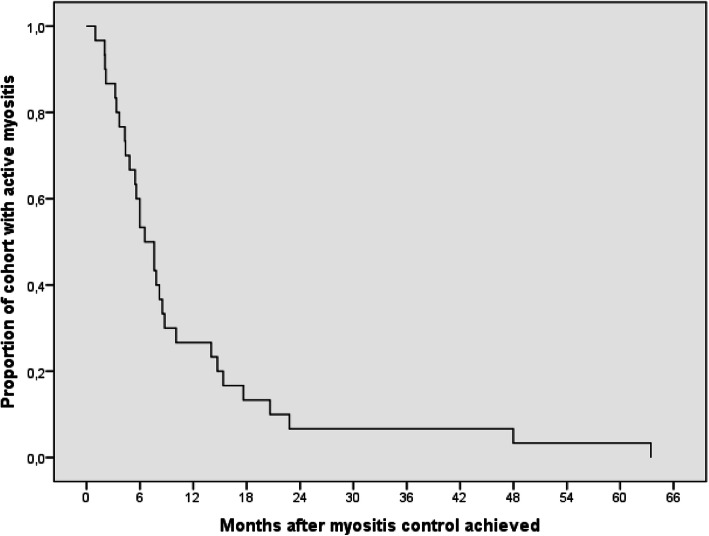
Time to myositis control. A Kaplan-Meier survival curve showing **t**he proportion of the cohort with control of myositis following initiation of treatment, displayed over time, in months. Patients who did not meet the outcome were censored after the time of last follow-up. Myositis control was obtained at a median of 7.1 months following initiation of treatment

**Fig. 2:**
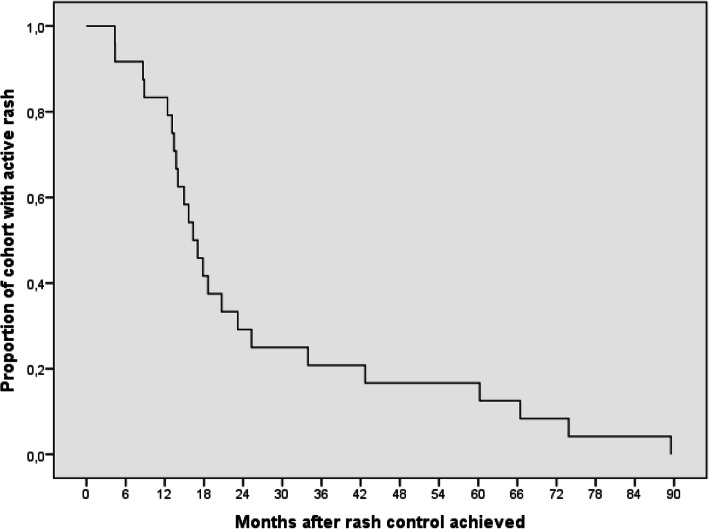
Time to cutaneous disease control. A Kaplan-Meier survival curve showing the proportion of the cohort with control of cutaneous disease following initiation of treatment, displayed over time, in months. Patients who did not meet the outcome were censored after the time of last follow-up. Cutaneous disease control was obtained at a median of 16.7 months

Myositis control was achieved within a median of 7.1 months (IQR 0.9–63.4), and only one patient had evidence of persistent muscle disease at the end of the study period. (Fig. 1). Cutaneous disease control was achieved within a median of 16.7 months (IQR 4.3–89.5) (Fig. 2). For seven patients (22%), rash never completely resolved. The median duration of oral prednisone treatment for the cohort was 9.1 months (IQR 4.7–17.4).

A monocyclic course of disease was observed in 10 patients (32%). In those patients, myositis control was reached in a median of 6.6 months (IQR 4.3–7.8) and cutaneous disease control was achieved in median 15.7 months (IQR 13.1–17). Oral prednisone was administered for a median of 8 months (IQR 4.5–8.8), and total treatment duration (all medications) was a median of 19.6 months (IQR 16.5–22.5). Eighteen patients (58%) followed a chronic continuous disease course. In those patients, myositis control was reached in a median 6 months (IQR 3.7–17.6) and cutaneous disease control in a median of 29.6 months (IQR 16.4–63.3). Oral prednisone was administered for a median of 9.9 months (IQR 7–30.4). Of those patients with chronic continuous disease, nine (50%) eventually achieved sustained disease control (complete disease control followed by medication withdrawal) by the end of the study period (Table 2). Finally, three patients (10%) were classified as having a polycyclic course, with symptom relapse occurring a median of 12 months after achieving sustained disease control. In two of these, complete disease control was again achieved after relapse, while one continued to have active disease at the end of the study period. Comparison between patients with monocyclic disease versus those with chronic continuous disease did not reveal a statistically significant difference in the time to myositis control, the dose or duration of steroid administration, the patient age at diagnosis, or the duration of untreated disease prior to diagnosis (Table 2). The time to cutaneous disease control was significantly shorter in the patients with monocyclic disease course, compared to those with chronic continuous disease course (*p* = 0.018; Table 2). Overall, sustained disease control was achieved in 21 patients (68%) by the end of the study period, with a median treatment duration of 22.3 months (IQR 18.0–31.4).

**Table 2:** Patient classification and time to treatment outcomes

	Monocyclic, *n* = 10	Chronic Continuous, *n* = 18	
Myositis control (months)	6.6 (4.3–7.8) (*n* = 10)	6 (3.7–17.6) (*n* = 17)	*p* = 0.334
Cutaneous disease control (months)	15.7 (13.1–17) (*n* = 9)	29.6, (16.4–63.3) (*n* = 12)	***p*** **= 0.018**
Duration of steroid use (months)	8 (4.5–8.8) (*n* = 9)	9.9 (7–30.4) (*n* = 18)	*p* = 0.145

Median value with interquartile range is shown for time to myositis control, cutaneous disease control, and duration of steroid use for JDM patients with monocyclic and chronic continuous disease courses. Statistical significance is denoted by a *p* value less than 0.05

Six patients (19%) had evidence of calcinosis during the study period. Calcinosis developed and resolved in five of the six patients (83%) during the study period. However, one patient, whose initial JDM symptoms were unrecognized for 18 months prior to presenting to our institution with extensive calcinosis but minimal other JDM symptoms beyond periungual telangiectasias, had persistent calcinosis. This patient was treated with diltiazem followed by pamidronate, which did not substantially improve the calcinosis by end of the study period.

### Growth

**Fig. 3:**
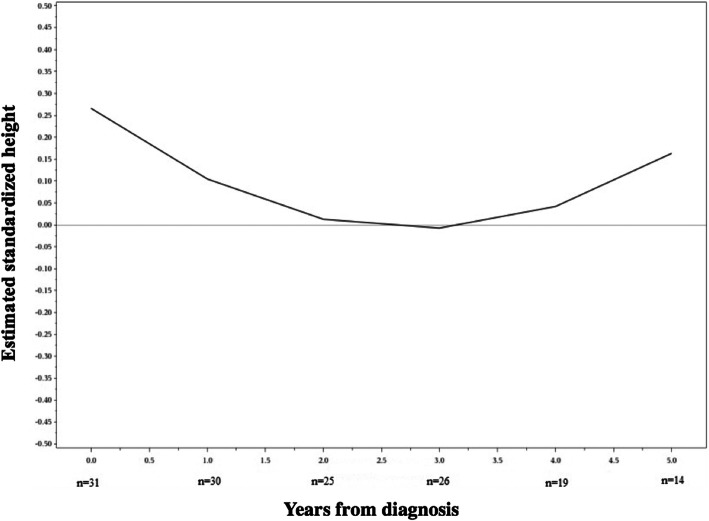
Standardized height during course of illness and treatment. The effect of disease and treatment from glucocorticoids on patients’ standardized height over years is shown by a random-effects mixed-model analysis. There was no statistically significant difference between the standardized height at the time of diagnosis and standardized height at year five

There was a statistically significant decrease compared to baseline (*p* = 0.02) in the quadratic patient height z-score, with the minimum values at 2.8 years after diagnosis. There was no statistically significant difference between the time of diagnosis and year five height (Fig. 3).

### Adverse events

Moderate and severe adverse events from treatment, including bone fractures, avascular necrosis and infections, are summarized in Table 3.

**Table 3:** Adverse effects observed in JDM cohort (*n* = 31)

Fractures (*n* = 4)	Talus (*n* = 1)
	Multiple thoracic vertebrae, T6-T10, T12 (*n* = 1)
	Avulsion fracture of the 3rd finger (*n* = 1)
	Distal tibia (following trauma) (*n* = 1)
Avascular necrosis (*n* = 2)	Knees bilaterally (*n* = 1) Ankle bones (*n* = 1)
Infections (*n* = 8) ^a^	RSV (*n* = 1)
	Campylobacter (*n* = 1)
	Oral thrush (*n* = 3)
	Cellulitis (*n* = 1)
	Pleural effusion (*n* = 1)
	Impetigo (*n* = 1)
	Vaginal candidiasis (*n* = 1)
	Viral meningitis (*n* = 1)
	Pneumonia (*n* = 1)
	Streptococcal bacteremia (*n* = 1)
	CMV reactivation (*n* = 1)
	Herpes zoster (*n* = 2)
Growth (*n* = 2)	Short stature (*n* = 1)
	Poor weight gain (*n* = 1)

^a^3 patients had multiple infections

## Discussion

In this single-center 14-year cohort study, we demonstrate that JDM patients treated with low-dose oral glucocorticoids (with IVMP only if dictated by disease severity) and early use of steroid-sparing treatments had comparable results to published outcomes in other treatment protocols advocating higher doses and longer duration of steroids, including a well-designed, similar sized, single-center cohort study in a timespan that partially overlapped with this study and used a more conventional glucocorticoid regimen [17]. Our initial treatment protocol included intravenous steroid use at diagnosis in only 38% of patients as a single short induction, contrasting with 84% in the comparator single-center cohort study, which also utilized continued weekly pulses following the induction [17]. Additionally, our treatment protocol employed lower starting doses of oral steroids (median dose of 0.85 mg/kg/day) compared to the more conventional higher dosing regimen of 2 mg/kg/day. Furthermore, there were differences in how glucocorticoids were weaned following the patient’s response, with the comparator study using a stated criteria of muscle enzyme level and muscle strength normalization before tapering, whereas we followed a broader assessment of overall clinical improvement [17]. Finally, although both advocated instituting additional therapies for continued disease activity, we utilized a higher frequency of IVIG (48% versus 20%) and rituximab (16% versus zero); however, this observation must take into account that the study periods do not completely overlap, and reports of rituximab use in JDM [27, 28] had not yet been published during the study period of the comparator study [17]. Our use of cyclophosphamide in 10% of patients compared to 4% in the comparator study [17] is an indirect assessment that both studies included patients with approximately similar disease severity, as cyclophosphamide is usually reserved for the most severely affected JDM patients. Another indirect severity measure is the occurrence of a monocyclic disease course, which occurred in 10 patients (32%) in our study and 18 patients (37%) in the comparator, both of which are consistent with other cohort frequencies. When comparing clinical responses to the different treatment regimens, our patients achieved myositis and cutaneous disease control with medians of 7.1 and 16.7 months, compared to myositis and cutaneous disease normalization medians of 13 and 19 months, respectively, in the comparator study, although the definitions used were slightly different. Sustained medication-free disease control in both cohorts was comparable, with 21 patients (68%) achieving medication-free disease control in our study, compared to 28 patients (57%) in the comparator study. Overall, it appears that outcome measures were comparable between the two studies.

Calcinosis is considered a marker of disease damage rather than an active disease feature itself [29, 30], although that understanding may be changing, as a recent survey of pediatric rheumatologists reported 73% of respondents considered the new development of calcinosis in a patient with absent muscle or skin disease as “active JDM disease” [31]. In our cohort, calcinosis developed in 19% of patients (6 out of 31 patients), but persisted in only one patient (3%). The calcinosis afflicting five of the six patients resolved with standard treatment prescribed for their JDM, without sequelae by the end of the of the study period. In the comparator study [17], six patients (12%) developed calcinosis, which was persistent in two patients (4%). The incidence and prevalence of calcinosis in the cohorts are comparable to the published literature [32], and if calcinosis is understood to be associated with prolonged or inadequately treated disease [33], then these findings also reflect the effectiveness of our treatment approach using lower doses and duration of glucocorticoids.

As described by Stringer *et. al.,* [18], the treatment strategies employed by pediatric rheumatologists in treating JDM are largely anchored around the use of high-dose oral glucocorticoids. This is echoed in the published treatment guidelines of CARRA [22] and SHARE [24] which both advocate for the use of IVMP and oral prednisone starting at 2 mg/kg/day, with relatively long tapers. At our center, patients were started at a lower dose of steroids (median 0.85 mg/kg/day), followed by a shorter taper based on the clinical and laboratory response of the individual patients with a complete taper off steroids in a median 9.1 months (IQR 4.7–17.4). High-dose IV methylprednisolone was reserved for moderate to severe JDM cases, comprising only 38% of patients. Our retrospective study suggests that lower steroid doses combined with universally early initiation of steroid-sparing agents led to lower cumulative glucocorticoid exposure and less associated adverse effects while retaining comparative favorable outcomes. A previous retrospective study of IVMP or high dose oral prednisone (5–30 mg/kg/day) therapy compared to “standard” oral prednisone (1–2 mg/kg/day) in JDM also found little difference in efficacy after controlling for disease severity [34].

We did not identify a significant difference in glucocorticoid usage between JDM patients with monocyclic and chronic continuous courses. While high-dose intravenous glucocorticoids remain the first-line option for patients with moderate to severe initial presentation, following this initial period, our data suggests that a taper beginning at a lower steroid doses can be used. Furthermore, our retrospective cohort suggests that sustainable results can be achieved without the need for frequent IVMP pulses following the induction IVMP pulse. In our study, steroid-sparing agents, particularly methotrexate, IVIG and hydroxychloroquine, were used universally early in the disease course and continued after glucocorticoid discontinuation. Monthly IVIG was used with very good results, despite the disadvantages of high cost and the need for an infusion facility. No major adverse events attributable to IVIG were seen in our cohort, possibly influenced by the intentional avoidance of central line placement.

Cutaneous disease is often more resistant than myositis to initial treatment with glucocorticoids and immunomodulators, persists for longer periods of time, and in some cases is refractory to multiple therapies. Hydroxychloroquine has been reported as an effective agent for refractory cutaneous disease [35, 36]⁠ and has been included by CARRA in consensus treatment plans of skin-predominant JDM [37]. In our study population, hydroxychloroquine was utilized in 13 patients with persistent skin involvement. Of the 15 cases in which IVIG was used, six cases were to treat refractory cutaneous disease, and it was effective in these patients. Four of the five patients in this cohort who received rituximab have been previously reported, and rituximab was found to be beneficial in three of four cases [27]. Mycophenolate mofetil has been reported to be a useful steroid-sparing agent in patients with JDM [38], and in this study cohort was used in 2 patients with beneficial results.

Limitations of the study include the retrospective design and the relatively modest number of subjects due to our applied exclusion criteria. Due to the retrospective nature of this study, we were not able to apply PRINTO’s criteria for inactive disease, which includes the childhood myositis assessment scale, manual muscle testing and/or physician global assessments. In reference to the comparator study, some definitions in the two studies were not identical, making some direct comparisons difficult. The lack of universal testing of myositis antibodies did not allow sufficient numbers to establish associations between specific antibodies and disease features or outcomes.

## Conclusion

Despite the limitations inherent in retrospective studies, this work provides evidence that lower oral corticosteroid dosing in combination with early use of steroid-sparing agents can achieve comparable outcomes to a similar retrospective work utilizing higher doses and longer duration of steroid therapy when assessed by rates of and time to muscle and skin disease control, rate of sustained disease control off medications, and occurrence of calcinosis. This approach limits the cumulative glucocorticoid exposure and potential long-term adverse events. This study complements several other prior retrospective studies [19–21] that also reported good outcomes with lower steroid dosing. However, a randomized control trial would be necessary to definitively determine if lower dose steroids are sufficient or if higher dose regiments are needed in JDM. As increasing numbers of other immunomodulatory agents are investigated in the treatment of JDM [39–41], ideally cumulative steroid exposure will continue to decrease.

## Data Availability

The datasets used and analyzed during the current study are available from the corresponding author on reasonable request.

## Abbreviations

JDM: Juvenile Dermatomyositis
IVMP: Intravenous pulse methylprednisolone
CARRA: Childhood Arthritis and Rheumatology Research Alliance
MRI: Magnetic resonance imaging
IVIG: Intravenous immune globulin
PRINTO: Paediatric Rheumatology International Trials Organization
